# Risk factors of suicide attempt among people with suicidal ideation in South Korea: a cross-sectional study

**DOI:** 10.1186/s12889-017-4491-5

**Published:** 2017-06-15

**Authors:** Soo Beom Choi, Wanhyung Lee, Jin-Ha Yoon, Jong-Uk Won, Deok Won Kim

**Affiliations:** 1 Department of Medical Engineering, Yonsei University College of Medicine, CPO Box, Seoul, 8044 South Korea; 2 Graduate Program in Biomedical Engineering, Yonsei University, Seoul, South Korea; 3 The Institute for Occupational Health, Yonsei University College of Medicine, Seoul, South Korea; 4 Graduate School of Public Health, Yonsei University College of Medicine, Seoul, South Korea; 5 Incheon Worker’s Health Center, Incheon, South Korea

**Keywords:** Suicide attempt, Suicidal ideation, Depression, Prediction model, Risk factor

## Abstract

**Background:**

Suicide is a serious public health concern worldwide, and the fourth leading cause of death in Korea. Few studies have focused on risk factors for suicide attempt among people with suicidal ideation. The aim of the present study was to investigate the risk factors and develop prediction models for suicide attempt among people with suicidal ideation in the Korean population.

**Method:**

This study included 1567 men and 3726 women aged 20 years and older who had suicidal ideation from the Korea National Health and Nutrition Examination Survey from 2007 to 2012. Among them, 106 men and 188 women attempted suicide. Multivariate logistic regression analysis with backward stepwise elimination was performed to find risk factors for suicide attempt. Sub-group analysis, dividing participants into under 50 and at least 50 years old was also performed.

**Results:**

Among people with suicidal ideation, age, education, cancer, and depressive disorder were selected as risk factors for suicide attempt in men. Age, education, national basic livelihood security, daily activity limitation, depressive disorder, stress, smoking, and regular exercise were selected in women. Area under curves of our prediction models in men and women were 0.728 and 0.716, respectively.

**Conclusions:**

It is important to pay attention to populations with suicidal ideation and the risk factors mentioned above. Prediction models using the determined risk factors could be useful to detect high-risk groups early for suicide attempt among people with suicidal ideation. It is necessary to develop specific action plans for these high-risk groups to prevent suicide.

**Electronic supplementary material:**

The online version of this article (doi:10.1186/s12889-017-4491-5) contains supplementary material, which is available to authorized users.

## Background

According to a World Health Organization (WHO) report, almost one million people died by suicide around the world in 2010 [1]. Suicide is a serious public health concern worldwide. Especially, in the Republic of Korea, suicidal deaths have increased since 1985, and the rate was over 30 per 100,000 persons in 2010 [2]. As a result, suicide was the fourth leading cause of death in Korea [3]. Furthermore, the age-standardized rate of suicide in Korea was 31.2 per 100,000 people, which is the highest among Organization for Economic Cooperation and Development (OECD) countries (11.3 per 100,000) [4].

Suicide is directly linked to suicide attempt resulting from a complex process including interacting biological, psychological, familial, socioeconomic status, and cultural circumstances [5]. Suicidal ideation, including both verbal and non-verbal manifestations, represents a clinical emergency in psychiatry [6]. Moreover, a previous study reported that individuals with suicidal ideation had a higher risk for suicide attempt than ones with non-suicidal ideation [7].

Therefore, finding the linkage between suicide attempt and suicidal ideation could be a key aspect of efficient suicide prevention. However, few studies have focused on risk factors for suicide attempt among people with suicidal ideation. Previous studies on suicide reported that suicidal ideation and suicide attempt were considered as a combined outcome [8, 9]. In addition, suicidal ideation was related with suicide processes [10].

Therefore, it could be helpful to understand associated suicide factors from ideation to attempt. The objective of the present study was to investigate the risk factors for suicide attempt among people with suicidal ideation, and develop prediction models for suicide attempt using logistic regression with the risk factors determined for the Korean population.

## Methods

### Study population

This study was conducted using data from the Korea National Health and Nutrition Examination Survey (KNHANES) conducted during 2007–2012. KNHANES is a nationwide representative survey of the health and nutritional status of the Korean population. The Health and Nutrition Survey division of the Korea Center for Disease Control and Prevention conducts this annual survey using a stratified and multistage probability sampling design to select household units, including non-institutionalized Korean civilians. As the data collection was performed by highly skilled surveyors and was controlled for quality, the data are considered highly accurate and reliable [11]. All KNHANES participants provided written consent to participate in the survey and for their personal data to be used.

Among the 50,405 subjects, 6358 subjects who were 20 years of age or older had suicidal ideation. After the exclusion of 1065 subjects with missing data or who did not answer questions on suicide attempt, 1567 men and 3726 women were finally included in the present study. The institutional review board of the Yonsei University Health System approved the protocol of this study (No. 4–2016-0872).

### Variables for suicide attempt

A structured interview about socioeconomic status, physical health, mental health, and health behaviors was performed by trained interviewers. Suicidal ideation and attempt were measured by responses to the following question: “During the past year, have you ever felt that you were willing to die?” Possible answer was never, rarely, yes, or always, and participants answering yes and always were categorized as having symptoms of suicidal ideation. Further, if the respondent indicated having symptoms of suicidal ideation, supplementary questions were asked: “Have you ever attempted suicide(s) in the past year?” These questions are simple but effective for detecting individuals vulnerable to suicide. Thus, previous research on suicidal risk used these questions to demonstrate risk for suicidal ideation and attempt [12, 13].

In this study, the variables of socioeconomic status for suicide included marital status, household income, living in rural area, employment status, education, and national basic livelihood security. Marital status was categorized as married, single, or divorced/separated/widowed. Household income was categorized according to quartiles of total income in the household. Living in rural area was based on whether the participant lives in a rural or urban area. Employment status was categorized as unemployed or employed. Education was categorized as above college, high, middle, and below elementary school. National basic livelihood security was defined as participation in a program, in which people who do not have enough income to meet their needs and those of their families in medical aid, housing, education benefits, etc., receive payment from the government. National basic livelihood security was categorized as never, ex-former, and current.

The variables for physical health included obesity, hypertension, diabetes mellitus, dyslipidemia, metabolic syndrome, cancer, daily activity limitation, and menopause. Subjects with one or more cancers of the stomach, liver, lung, colon, cervix, or breast were defined as “major cancer” patients based on their answering “yes” to the question “Were you diagnosed with cancer(s) by a physician? [12]” Daily activity limitation was defined as occurring when participants have limitations in activities of daily living due to health problems (fracture or articular injury, other injury, arthritis or rheumatism, cardiac disorders, pulmonary disorders or asthma, stroke, diabetes, hypertension, neck or back disorders, cancers, oral or dental disorders, ophthalmic disorders, auditory disorders, dementia, depressive, anxiety, or mental disorders, mental retardation, obesity, ageing, etc.).

The variables for mental health included depressive disorder and stress. Depressive disorder was defined by the question "Have you been diagnosed with depressive disorder by a physician?" or whether the participants experienced depressive mood for two or more continuous weeks. Stress was categorized as minimally, moderately, and extremely stressful.

The risk factors of health behaviors relevant for suicide included drinking, smoking, and regular exercise. Drinking was categorized as never, normal, and risky drinking. Risky drinking was defined as drinking more than seven glasses of alcohol twice per week in men and more than five glasses of alcohol twice per week in women. Smoking was categorized as never, ex-former, and current. Regular exercise was categorized as hardly ever, regular moderate, and strenuous (at least three times per week for at least 20 min of aerobic activity at a time) [12].

### Statistical analyses

The characteristics of the participants were reported as means (standard deviation [SD]) for continuous variables, and as numbers (%) for categorical variables. In order to evaluate the association between each of these variables and suicide attempt, an independent samples t-test was performed for each continuous variable and a chi-square test was performed for each categorical variable (Tables 1 and 2). Multivariate logistic regression analysis with backward stepwise elimination was also performed to find risk factors for suicide attempt with a threshold of *p* = 0.05 after gender stratification. Additionally, sub-group analysis, depending on whether participants were under 50 or at least 50 years old (Tables 3 and 4), was performed because the mean age in this study population was in the early fifties. Spearman’s rank correlation analysis was conducted for correlations between the selected variables by logistic regression analysis (Additional file 1: Tables S1 and S2).

**Table 1:** Demographic and clinical characteristics of male participants, and the association between these characteristics and suicide attempt (*n* = 1567)

	Suicide attempt		*P* ^a^
	No (*n* = 1461)	Yes (*n* = 106)	
Age (years)	54.8 ± 16.6	56.9 ± 15.8	0.216
Height (cm)	168.0 ± 7.1	166.6 ± 6.2	0.049*
Weight (kg)	66.9 ± 11.5	64.5 ± 10.2	0.036*
Marital status (n, %)			0.112
Married	1115 (76.3)	75 (70.8)	
Single	215 (14.7)	15 (14.2)	
Divorced/separated/widowed	131 (9.0)	16 (15.1)	
Household income (n, %)			0.017*
Low	458 (31.3)	49 (46.2)	
Moderate–low	422 (28.9)	22 (20.8)	
Moderate–high	306 (20.9)	19 (17.9)	
High	275 (18.8)	16 (15.1)	
Rural area (n, %)	426 (29.2)	32 (30.2)	0.822
Employment status (n, %)	930 (63.7)	56 (52.8)	0.026*
Education (n, %)			<0.001*
Above college	327 (22.4)	8 (7.5)	
High school	448 (30.7)	24 (22.6)	
Middle school	196 (13.4)	25 (23.6)	
Below elementary school	490 (33.5)	49 (46.2)	
National basic livelihood security (n, %)			0.008*
Never	1300 (89.0)	84 (79.2)	
Ex-former	63 (4.3)	10 (9.4)	
Current	98 (6.7)	12 (11.3)	
Obesity (n, %)			0.210
Normal	925 (63.3)	73 (68.9)	
Underweight	67 (4.6)	7 (6.6)	
Overweight	469 (32.1)	26 (24.5)	
Hypertension (n, %)			0.460
Normal	478 (32.7)	30 (28.3)	
Prehypertension	395 (27.0)	27 (25.5)	
Hypertension	588 (40.2)	49 (46.2)	
Diabetes (n, %)			0.798
Normal	897 (61.4)	63 (59.4)	
Prediabetes	331 (22.7)	27 (25.5)	
Diabetes	233 (15.9)	16 (15.1)	
Dyslipidemia (n, %)	520 (35.6)	33 (31.1)	0.354
Metabolic syndrome (n, %)	399 (27.3)	29 (27.4)	0.991
Cancer ^b^ (n, %)	54 (3.7)	10 (9.4)	0.004*
Daily activity limitation	455 (31.1)	49 (46.2)	0.001*
Depressive disorder (n, %)	747 (51.1)	83 (78.3)	<0.001*
Stress (n, %)			0.212
Minimally stressful	124 (8.5)	10 (9.4)	
Moderately	593 (40.6)	32 (30.2)	
Stressful	550 (37.6)	47 (44.3)	
Extremely stressful	194 (13.3)	17 (16.0)	
Drinking (n, %)			0.195
Never	308 (21.1)	28 (26.4)	
Normal drinking	833 (57.0)	51 (48.1)	
Risky drinking	320 (21.9)	27 (25.5)	
Smoking (n, %)			0.192
Never	186 (12.7)	20 (18.9)	
Ex-former	279 (19.1)	18 (17.0)	
Current	996 (68.2)	68 (64.2)	
Regular exercise (n, %)			0.696
Hardly ever	981 (67.1)	72 (67.9)	
Regular moderate	413 (28.3)	31 (29.2)	
Strenuous	67 (4.6)	3 (2.8)	

*p < 0.05

^a^P-values of continuous and binary variables were calculated using the independent samples *t*-test and chi-square test, respectively

^b^Subjects with one or more cancers of the stomach, liver, lung, colon, cervix, or breast

**Table 2:** Demographic and clinical characteristics of female participants, and the association between these characteristics and suicide attempt (*n* = 3726)

	Suicide attempt		*P* ^a^
	No (*n* = 3538)	Yes (*n* = 188)	
Age (years)	54.4 ± 17.6	51.2 ± 17.7	0.016*
Height (cm)	155.0 ± 7.0	155.5 ± 7.2	0.377
Weight (kg)	57.0 ± 9.9	58.1 ± 11.4	0.178
Marital status (n, %)			0.130
Married	2222 (62.8)	106 (56.4)	
Single	357 (10.1)	26 (13.8)	
Divorced/separated/widowed	959 (27.1)	56 (29.8)	
Household income (n, %)			0.007*
Low	1167 (33.0)	75 (39.9)	
Moderate–low	949 (26.8)	50 (26.6)	
Moderate–high	792 (22.4)	47 (25.0)	
High	630 (17.8)	16 (8.5)	
Rural area (n, %)	998 (28.2)	49 (26.1)	0.524
Employment status (n, %)	1514 (42.8)	68 (36.2)	0.073
Education (n, %)			0.129
Above college	580 (16.4)	19 (10.1)	
High school	889 (25.1)	52 (27.7)	
Middle school	344 (9.7)	22 (11.7)	
Below elementary school	1725 (48.8)	95 (50.5)	
National basic livelihood security (n, %)			<0.001*
Never	3127 (88.4)	144 (76.6)	
Ex-former	144 (4.1)	13 (6.9)	
Current	267 (7.5)	31 (16.5)	
Obesity (n, %)			0.777
Normal	2157 (61.0)	116 (61.7)	
Underweight	214 (6.0)	9 (4.8)	
Overweight	1167 (33.0)	63 (33.5)	
Hypertension (n, %)			0.232
Normal	1549 (43.8)	90 (47.9)	
Prehypertension	660 (18.7)	39 (20.7)	
Hypertension	1329 (37.6)	59 (31.4)	
Diabetes (n, %)			0.935
Normal	2563 (72.4)	134 (71.3)	
Prediabetes	531 (15.0)	29 (15.4)	
Diabetes	444 (12.5)	25 (13.3)	
Dyslipidemia (n, %)	1524 (43.1)	80 (42.6)	0.888
Metabolic syndrome (n, %)	1073 (30.3)	52 (27.7)	0.437
Cancer ^b^ (n, %)	164 (4.6)	7 (3.7)	0.720
Daily activity limitation (n, %)	1085 (30.7)	84 (44.7)	<0.001*
Menopause (n, %)			0.067
Ongoing	1412 (39.9)	87 (46.3)	
Post	1890 (53.4)	95 (50.5)	
Artificial	236 (6.7)	6 (3.2)	
Depressive disorder (n, %)	2276 (64.3)	167 (88.8)	<0.001*
Stress (n, %)			<0.001*
Minimally stressful	274 (7.8)	5 (2.7)	
Moderately	1258 (35.6)	34 (18.1)	
Stressful	1508 (42.7)	90 (47.9)	
Extremely stressful	494 (14.0)	59 (31.4)	
Drinking (n, %)			<0.001*
Never	1536 (43.4)	71 (37.8)	
Normal drinking	1836 (51.9)	93 (49.5)	
Risky drinking	166 (4.7)	24 (12.8)	
Smoking (n, %)			<0.001*
Never	2964 (83.8)	127 (67.6)	
Ex-former	126 (3.6)	8 (4.3)	
Current	448 (12.7)	53 (28.2)	
Regular exercise (n, %)			<0.001*
Hardly ever	2715 (76.7)	130 (69.1)	
Regular moderate	693 (19.6)	38 (20.2)	
Strenuous	130 (3.7)	20 (10.6)	

*p < 0.05

^a^P-values of continuous and binary variables were calculated using the independent samples *t*-test and chi-square test, respectively

^b^Subjects with one or more cancers of the stomach, liver, lung, colon, cervix, or breast

**Table 3:** Results of multivariate logistic regression analysis with backward stepwise elimination for prediction of suicide attempts in men for different age groups

	Odds ratio (95% Confidence Interval)		
	All (*n* = 1567)	Age < 50 (*n* = 593)	Age > = 50 (*n* = 974)
Suicide attempt (n, %)	106 (6.8%)	34 (5.7%)	72 (7.4%)
Age	0.981 (0.964–0.997)		
Education			
Above college	Reference	Reference	Reference
High school	2.130 (0.940–4.826)	3.858 (1.269–11.728)	0.920 (0.248–3.414)
Middle school	6.301 (2.673–14.856)	8.082 (2.098–31.138)	3.270 (1.052–10.166)
Below elementary school	5.803 (2.461–13.680)	17.760 (4.123–76.506)	2.513 (0.859–7.347)
Cancer ^a^	2.401 (1.136–5.074)		2.470 (1.162–5.248)
Daily activity limitation		2.446 (1.058–5.652)	
Depressive disorder	3.274 (2.031–5.279)	3.007 (1.316–6.870)	3.468 (1.895–6.350)
Drinking			
Never		Reference	
Normal drinking		4.111 (0.507–33.347)	
Risky drinking		8.517 (1.018–71.242)	
Smoking			
Never			Reference
Ex-former			0.475 (0.223–1.009)
Current			0.429 (0.227–0.811)

^a^Subjects with one or more cancers of the stomach, liver, lung, colon, cervix, or breast

**Table 4:** Results of multivariate logistic regression analysis with backward stepwise elimination for prediction of suicide attempts in women for different age groups

	Odds ratio (95% Confidence Interval)		
	All (*n* = 3726)	Age < 50 (*n* = 1402)	Age > = 50 (*n* = 2136)
Suicide attempt (n, %)	188 (5.1%)	92 (6.6%)	96 (4.5%)
Age	0.969 (0.955–0.983)		
Education			
Above college	Reference	Reference	Reference ^a^
High school	1.715 (0.987–2.979)	1.624 (0.923–2.858)	
Middle school	2.422 (1.223–4.795)	2.338 (1.087–5.032)	1.669 (0.558–4.993)
Below elementary school	3.797 (1.929–7.473)	3.383 (1.519–7.536)	2.799 (1.177–6.658)
National basic livelihood security			
Never	Reference	Reference	
Ex-former	1.822 (0.985–3.368)	2.802 (1.082–7.256)	
Current	1.820 (1.169–2.833)	2.426 (1.233–4.776)	
Daily activity limitation	1.641 (1.166–2.308)		
Depressive disorder	3.099 (1.930–4.976)	3.504 (1.712–7.170)	3.026 (1.609–5.690)
Stress			
Minimally stressful	Reference	Reference	Reference
Moderately	1.210 (0.461–3.175)	0.749 (0.091–6.174)	1.683 (0.567–4.997)
Stressful	1.957 (0.768–4.986)	1.760 (0.227–13.635)	2.466 (0.858–7.082)
Extremely stressful	3.555 (1.370–9.222)	3.700 (0.470–29.156)	4.103 (1.373–12.262)
Smoking			
Never	Reference	Reference	Reference
Ex-former	1.215 (0.560–2.634)	1.192 (0.450–3.152)	1.463 (0.420–5.094)
Current	2.264 (1.580–3.245)	2.108 (1.300–3.418)	2.712 (1.591–4.625)
Regular exercise			
Hardly ever	Reference		Reference
Regular moderate	1.093 (0.743–1.608)		1.124 (0.637–1.985)
Strenuous	3.430 (1.991–5.911)		6.857 (3.276–14.356)

^a^There was no subject with education above college and suicide attempts

The data set was divided randomly into two independent training and validation groups to test for internal validation. The training group, comprising 70% (1097 men with 79 suicide attempts and 2608 women with 135 suicide attempts) of the entire dataset, was used to construct a logistic regression model. The validation group, comprising 30% (470 men with 27 suicide attempts and 1118 women with 53 suicide attempts) of the entire dataset, was used to assess the performance of the model for suicide attempt. Receiver operating characteristic (ROC) curve and area under the curve (AUC) analysis were executed to verify the performance of logistic regression models for men and women. All statistical analyses were two-sided and executed using SPSS 23.0 (IBM Corp., Armonk, NY). A *p*-value <0.05 was considered statistically significant.

## Results

### Baseline characteristics

Table 1 lists the demographic and clinical characteristics of the study population in men. In the results of independent samples t-tests and the chi-square tests, suicide attempt was significantly correlated with the nine variables marked with an *, which were height, weight, household income, employment status, education, national basic livelihood security, cancer, daily activity limitation, and depressive disorder. Table 2 lists the demographic and clinical characteristics of the study population in women. Suicide attempt was significantly correlated with the nine variables marked with an *, which were age, household income, national basic livelihood security, daily activity limitation, depressive disorder, stress, drinking, smoking, and regular exercise.

### Risk factors and prediction models for suicide attempt among participants with suicidal ideation

In Table 3, age, education, cancer, and depressive disorder were selected by multivariate logistic regression analysis for men. Age was negatively associated with suicide attempt among all the participants, and younger men were more vulnerable to suicide attempt than older men. When the above college group was used as the reference group, the middle school and below elementary school groups had odds ratios (ORs) of 6.301 (the largest) and 5.803, respectively, but the high school group was not significant. Cancer and depressive disorder had ORs of 2.401 and 3.274, respectively. Age-group differences were observed in men. Educational level and depressive disorder were predictors of suicide attempt in both age-groups. However, daily activity limitation and drinking alcohol were predictors only in the under 50 years old group. Cancer and smoking were predictors only in the over 50 years old group.

In Table 4, age, education, national basic livelihood security, daily activity limitation, depressive disorder, stress, smoking, and regular exercise were selected for women. Age was negatively associated with suicide attempt, and younger women were more vulnerable to suicide attempt than older women. When the above college group was used as the reference group, the middle school and below elementary school groups had ORs of 2.422 and 3.797 (which was the largest), respectively, but the high school group was not significant, the same as for men. Subjects who received national basic livelihood security at that time had an OR of 1.820. Daily activity limitation and depressive disorder had ORs of 1.641 and 3.099, respectively. Extremely stressful participants had an OR of 3.555. Current smokers had an OR of 2.264. Participants who did strenuous exercise had an OR of 3.430, which was not expected. There were differences in the predictors between age-groups. National basic livelihood security was significantly associated with suicidal attempt among younger-aged women. Daily activity limitation was not a predictor in either age group. Regular exercise level was a predictor only in the over 50 years old group in women.

**Fig. 1:**
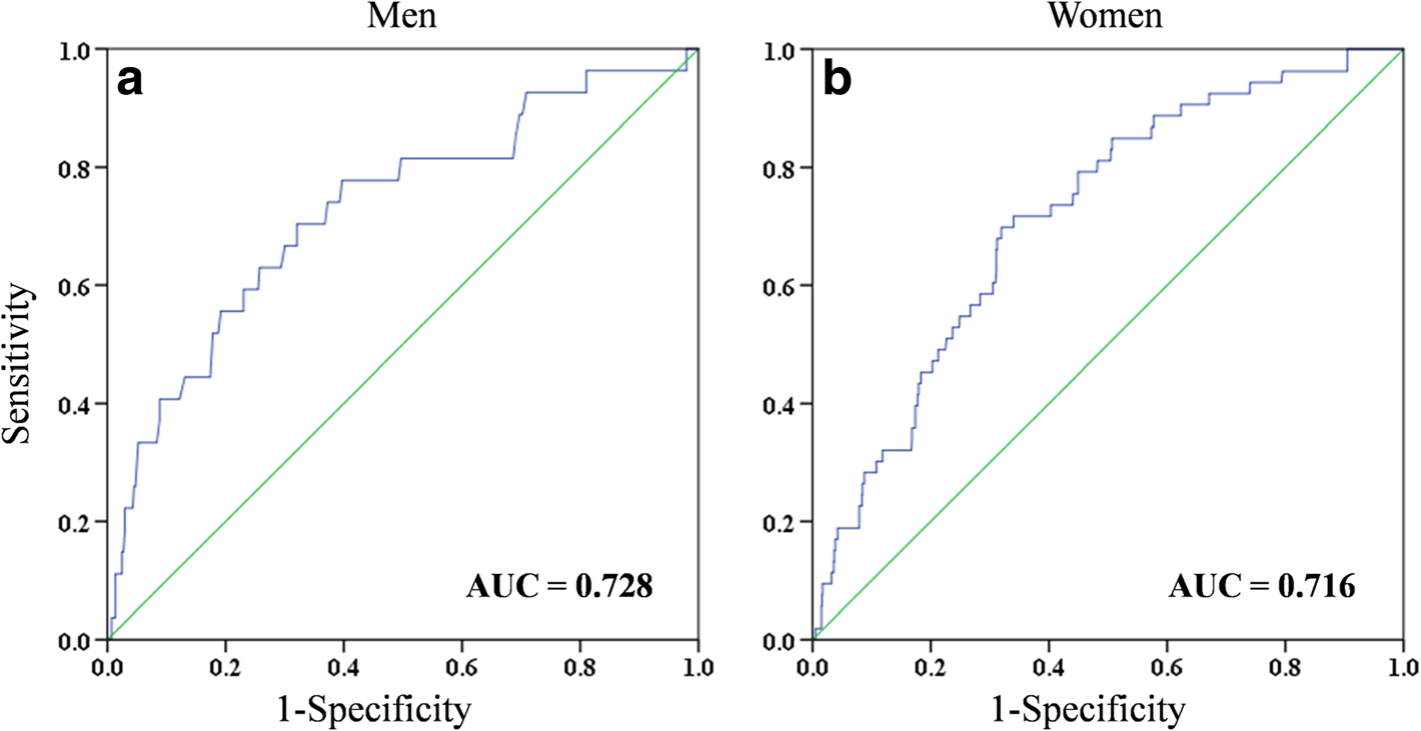
Receiver operating characteristic curves and area under the curve (AUC) for suicide attempt in men (**a**) and women (**b**)

For both men and women, age, low education level, and depressive disorder were common risk factors for suicide attempt. However, cancer was not significant in women, and national basic livelihood security, daily activity limitation, stress, smoking, and regular exercise were not significant in men. Fig. 1 (a) and (b) demonstrate the performance of our logistic regression models for prediction of suicide attempt in men and women. The AUCs of logistic regression were 0.728 and 0.716, respectively for men and women.

## Discussion

In this study, the constructed multiple logistic regression models determined the risk factors for suicide attempt among people with suicidal ideation. Age, education, cancer, and depressive disorder were selected for men. Age, education, national basic livelihood security, daily activity limitation, depressive disorder, stress, smoking, and regular exercise were selected for women. The logistic regression models effectively predicted suicide attempt from the analysis.

These findings could help us understand the nature of suicide for men and women. For both men and women, depressive disorder has a key role in suicide attempt (OR: 3.274 for men and 3.099 for women). These results are in line with those of previous studies. In 2015, the American Foundation for Suicide Prevention indicated depression as a risk factor and a warning sign for suicide [14]. A current report from WHO states that mental disorders, especially depression, are closely related with suicide [15]. The association of depression with suicide may be explained by the bio-mechanisms of the nervous system. Depression patients have disrupted central 5-hydroxytryptamine (5-HT) metabolism, which could result in 5-HT deficiency and serotonergic hypoactivity [16]. This interrupted 5-HT metabolism is also closely related with increased risk of suicide [17]. According to a previous review study, depression is strongly associated with suicide including suicidal behavior [18]. Thus, this study also suggests that depression could increase risk for suicide attempt from suicidal ideation.

This study also demonstrated low education level as the highest risk factor for suicide attempt among those with suicidal ideation for both men (OR: 6.301 for middle school) and women (OR: 3.797 for below elementary school) in Tables 3 and 4. In a previous multi-nation study, low education level was linked to increased risk for suicide [19]. In addition, short educational duration was significantly linked to suicide in a well-established cohort study [20]. Therefore, more attention should be paid to males and females with suicidal ideation who have low levels of education.

The current investigation suggested gender differences between men and women for risk factors of suicide attempt from ideation. Male participants over 50 years old with a diagnosed cancer showed significant increased risk for suicide attempt. The previous study also reported an approximately two-times higher incidence of suicide in individuals who had a cancer diagnosis than the general population, especially male cancer survivors [20]. It was also reported that increased severity of the cancer was linked to increased suicide risk [21]. Furthermore, there was an increased risk for death by suicide among men with even low-risk cancer [22].

Female participants with low socio-economic status have a significantly higher number of suicide attempts, especially those who are receiving national basic livelihood security. Low financial status, such that it is difficult to live without a supplementary living allowance, may exacerbate any ongoing deterioration in psychological well-being, and eventually leads to suicidal ideation among older adults in Australia and Korea [23, 24]. Moreover, other research has shown that low socio-economic status characteristics are directly associated with risks of suicide attempt [25]. It is possible that low socio-economic status increased the risk of suicidal ideation for both men and women, but women who suffer from the hardships of life were more vulnerable to suicide attempt than men as shown in our study (OR: 1.820). National basic livelihood security has been considered as a minimum social safety net. Our study could indicate that suicide prevention professionals should pay attention to women under 50 years old who are receiving national basic livelihood security. Therefore, the function and limitations of national basic livelihood security should be improved.

Activity limitation in female participants was significantly associated with suicide attempt among those with suicidal ideation. Male participants only under 50 years old with daily activity limitation were vulnerable to suicide attempt. It was reported that limitation in activities of daily living is independently associated with the presence of suicidal ideation, particularly in older people [26]. Moreover, women with severe stress (OR: 3.555) and suicidal ideation were more vulnerable to suicide attempt than men in our study. It is well-known that stress is related to suicide. The previous study also reported that women students showed higher levels of stress and depressive symptoms than men [27].

Smoking and risky drinking have been identified as risk factors for suicide [28]. A previous study demonstrated that frequent drinking was associated with suicide attempt in females, but not in males [29]. However, not frequent drinking but smoking was a risk factor for women with suicidal ideation in our study. Smoking and risky drinking have been correlated with psychiatric disorders [30]. Women with suicidal ideation and smoking (OR: 2.264) could be more vulnerable to suicide attempt than men. Therefore, more attention needs to be paid to women with suicidal ideation who smoke to prevent suicide. The present study showed among female participants, strenuous exercise was significantly associated with suicide attempt (OR: 3430). This result is different from a previous study and guideline for depressive disorder [31]. Further study and analysis of the relationship between strenuous exercise and suicide attempt are warranted.

A previous study about the lifetime prevalence of suicide attempts among people with suicidal ideation either with or without a plan demonstrated that being female, previously married, aged less than 25 years, having low education, and mental disorder significantly increased risks for suicide attempt among people with suicidal ideation [32]. These results, except previously married status, supported our selected risk factors for both males and females in this study. Although the previous study was a cohort study and systematically analyzed, our study demonstrated additional risk factors such as cancer, low socio-economic status, activity limitation, stress, and smoking, which were not included in their previous study. The additional risk factors could be a result of characteristics specific to Korean people. Moreover, our study suggested prediction models for suicide attempt.

This study is limited by the lack of information on death certification. Thus, we investigated the association between suicidal ideation and failed suicide attempt. Second, this was a cross-sectional study, which limits attributions about the direction of causality between some variables, such as smoking, exercise, and stress. Third, there could be uncertainty about the reliability and validity of answers from the questionnaires for suicidal ideation and attempt, because the questionnaires involved sensitive issues. However, questionnaires about suicidal ideation and attempt were considered important tools to screen for the population that is vulnerable to suicide, worldwide. Lastly, we performed the aged sub-group analysis only with cutoff point of 50 age, because of the relatively small number of people with suicide attempts in this study. However, risk factors for suicide attempts could be different for people with cutoff points of 20’s and 40’s etc.

## Conclusions

Suicide is largely preventable with suitable action and plans. According to a WHO report, identifying groups vulnerable to suicide could be helpful to prevent loss of life by suicide [1]. It is important to pay attention to populations with suicidal ideation and several risk factors: age, low education, and depressive disorder, which are applicable to both male and female populations; males with cancer diagnosis; females with low economic status; limitations in activities of daily living; heavy stress; and smoking. Prediction models using the determined risk factors could be useful for early detection of high-risk groups for suicide attempt among people with suicidal ideation. Moreover, it is necessary to develop specific action plans for these high-risk groups to prevent suicide.

## Additional file

Additional file 1: Table S1. Correlation coefficients between suicide attempts and the selected variables by logistic regression analysis using Spearman’s rank correlation analysis in men. **Table S2.** Correlation coefficients between suicide attempts and the selected variables by logistic regression analysis using Spearman’s rank correlation analysis in women. (DOC 39 kb)

## Abbreviations

5-HT: 5-hydroxytryptamine
AUCs: Area under curves
KNHANES: Korea National Health and Nutrition Examination Survey
ROC: Receiver operating characteristic
SD: Standard deviation
WHO: World Health Organization
