# Supplementary material for: Risk factors of suicide attempt among people with suicidal ideation in South Korea: a cross-sectional study
Source: BMC Public Health. 2017 Jun 15;17:579. doi: 10.1186/s12889-017-4491-5 (PMC5472995; doi:10.1186/s12889-017-4491-5)
Supplement: Additional file 1: Table S1. — Correlation coefficients between suicide attempts and the selected variables by logistic regression analysis using Spearman’s rank correlation analysis in men. Table S2. Correlation coefficients between suicide attempts and the selected variables by logistic regression analysis using Spearman’s rank correlation analysis in women. (DOC 39 kb) [file 12889_2017_4491_MOESM1_ESM.doc]

**Supplementary Table 1** Correlation coefficients between suicide attempts and the selected variables by logistic regression analysis using Spearman's rank correlation analysis in men

|  | Education | Cancer | Depressive disorder | Suicide attempts |
| --- | --- | --- | --- | --- |
| Age | - 0.609** | 0.169** | 0.058* | 0.032 |
| Education |  | - 0.086** | - 0.043 | - 0.108** |
| Cancer |  |  | 0.065** | 0.073** |
| Depressive disorder |  |  |  | 0.137** |

* *p*-value < 0.05, ** *p*-value < 0.01

**Supplementary Table 2** Correlation coefficients between suicide attempts and the selected variables by logistic regression analysis using Spearman's rank correlation analysis in women

|  | Education | National basic livelihood security | Daily activity limitation | Depressive disorder | Stress | Smoking | Regular exercise | Suicide attempts |
| --- | --- | --- | --- | --- | --- | --- | --- | --- |
| Age | -0.776** | 0.146** | 0.366** | -0.015 | -0.167** | -0.121** | -0.177** | -0.040* |
| Education |  | -0.176** | -0.343** | -0.015 | 0.107** | 0.070** | 0.157** | -0.020 |
| National basic livelihood security |  |  | 0.166** | 0.044** | 0.026 | 0.071** | -0.047** | 0.080** |
| Daily activity limitation |  |  |  | 0.094** | 0.060** | -0.028 | -0.078** | 0.066** |
| Depressive disorder |  |  |  |  | 0.252** | 0.024 | 0.051** | 0.113** |
| Stress |  |  |  |  |  | 0.052** | 0.044** | 0.121** |
| Smoking |  |  |  |  |  |  | -0.011 | 0.097** |
| Regular exercise |  |  |  |  |  |  |  | 0.046** |

* *p*-value < 0.05, ** *p*-value < 0.01
